# Dual-energy computed tomography iodine quantification combined with laboratory data for predicting microvascular invasion in hepatocellular carcinoma: a two-centre study

**DOI:** 10.1093/bjr/tqae116

**Published:** 2024-06-13

**Authors:** Huan Li, Dai Zhang, Jinxia Pei, Jingmei Hu, Xiaohu Li, Bin Liu, Longsheng Wang

**Affiliations:** Department of Radiology, The Second Affiliated Hospital of Anhui Medical University, Hefei, Anhui 230601, China; Medical Imaging Research Center, Anhui Medical University, Hefei, Anhui 230601, China; Department of Radiology, The Second Affiliated Hospital of Anhui Medical University, Hefei, Anhui 230601, China; Medical Imaging Research Center, Anhui Medical University, Hefei, Anhui 230601, China; Department of Radiology, The Second Affiliated Hospital of Anhui Medical University, Hefei, Anhui 230601, China; Medical Imaging Research Center, Anhui Medical University, Hefei, Anhui 230601, China; Department of Radiology, The Second Affiliated Hospital of Anhui Medical University, Hefei, Anhui 230601, China; Medical Imaging Research Center, Anhui Medical University, Hefei, Anhui 230601, China; Department of Radiology, The First Affiliated Hospital of Anhui Medical University, Hefei, Anhui 230601, China; Department of Radiology, The First Affiliated Hospital of Anhui Medical University, Hefei, Anhui 230601, China; Department of Radiology, The Second Affiliated Hospital of Anhui Medical University, Hefei, Anhui 230601, China; Medical Imaging Research Center, Anhui Medical University, Hefei, Anhui 230601, China

**Keywords:** hepatocellular carcinoma, microvascular invasion, dual-energy computed tomography, principal component analysis

## Abstract

**Objectives:**

Microvascular invasion (MVI) is a recognized biomarker associated with poorer prognosis in patients with hepatocellular carcinoma. Dual-energy computed tomography (DECT) is a highly sensitive technique that can determine the iodine concentration (IC) in tumour and provide an indirect evaluation of internal microcirculatory perfusion. This study aimed to assess whether the combination of DECT with laboratory data can improve preoperative MVI prediction.

**Methods:**

This retrospective study enrolled 119 patients who underwent DECT liver angiography at 2 medical centres preoperatively. To compare DECT parameters and laboratory findings between MVI-negative and MVI-positive groups, Mann-Whitney *U* test was used. Additionally, principal component analysis (PCA) was conducted to determine fundamental components. Mann-Whitney *U* test was applied to determine whether the principal component (PC) scores varied across MVI groups. Finally, a general linear classifier was used to assess the classification ability of each PC score.

**Results:**

Significant differences were noted (*P *<* *.05) in alpha-fetoprotein (AFP) level, normalized arterial phase IC, and normalized portal phase IC between the MVI groups in the primary and validation datasets. The PC1-PC4 accounted for 67.9% of the variance in the primary dataset, with loadings of 24.1%, 16%, 15.4%, and 12.4%, respectively. In both primary and validation datasets, PC3 and PC4 were significantly different across MVI groups, with area under the curve values of 0.8410 and 0.8373, respectively.

**Conclusions:**

The recombination of DECT IC and laboratory features based on varying factor loadings can well predict MVI preoperatively.

**Advances in knowledge:**

Utilizing PCA, the amalgamation of DECT IC and laboratory features, considering diverse factor loadings, showed substantial promise in accurately classifying MVI. There have been limited endeavours to establish such a combination, offering a novel paradigm for comprehending data in related research endeavours.

## Introduction

Hepatocellular carcinoma (HCC) is the sixth most common cancer worldwide and the second leading cause of cancer mortality.^1^ MVI primarily refers to the observation of cancer cell nests in the vascular lumen lined with endothelial cells under a microscope, where cancer cells invade surrounding tissues and enter the vasculature to develop intrahepatic or extrahepatic metastasis.^2^ Patients with microvascular invasion (MVI) in HCC usually present with higher postoperative recurrence rates and lower 5-year relative survival rates.^2^^,^^3^ Therefore, accurate preoperative MVI prediction in HCC is essential for effective treatment planning and prognosis of patients.^4^

Several morphological features in preoperative imaging, including the hypodense halo sign, visible intratumoural arteries, and tumour shape, are associated with MVI.^5–7^ However, the use of one or 2 morphological features to establish MVI diagnosis is difficult and inaccurate. Therefore, it is important to improve diagnostic accuracy by combining quantitative parameters with morphological features.^8–11^ Despite several efforts to predict the risk of MVI using CT/MRI multiphase-enhanced and diffusion-weighted images combined with clinical and laboratory results,^8^^,^^12–14^ the clinical use of radiomics remains challenging.^15^^,^^16^ Dual-energy CT (DECT) can improve material differentiation using 2 different X-ray energy spectra, which provide more information on energy-dependent specific materials, particularly iodine. Iodine concentration (IC) provides valuable information on tissue blood supply and can be used to classify preoperative MVI.^17^^,^^18^ To our knowledge, no prior research has explored the use of DECT iodine quantification combined with laboratory data to predict preoperative MVI risk.

Therefore, this study aimed to determine whether the combination of preoperative contrast DECT-based IC measurements with laboratory results may improve preoperative MVI prediction. Considering the redundant information between different measurements, principal component analysis (PCA) was performed to reveal the underlying fundamental components. Moreover, to quantify the MVI predictive ability, a general linear classifier was applied to each fundamental component of the primary and validation datasets.

## Methods

### Patients

This retrospective study was conducted using data from 2 medical centres. The institutional review boards of both medical centres approved this study. Informed consent was waived owing to the retrospective nature of the study.

A total of 119 patients who were postoperatively diagnosed with HCC and satisfied the following inclusion criteria were included in the analysis: (1) availability of complete preoperative clinical and contrast-enhanced DECT data, (2) interval between CT examination and the blood samples for laboratory analysis and resection should be <1 week to avoid lesion changes that may affect the results, and (3) no preoperative treatment and subsequent underwent tumour resection at 2 medical centres. The exclusion criteria were as follows: (1) the presence of multiple lesions and (2) definite macrovascular invasion. To obtain and verify the fundamental components, primary and validation datasets were established. The primary dataset included 82 patients from centre 1 (The Second Affiliated Hospital of Anhui Medical University, Hefei), whereas the validation dataset included 37 patients from centre 2 (The First Affiliated Hospital of Anhui Medical University, Hefei). All patients underwent resection at centres 1 and 2 from June 1, 2018, to October 30, 2022.

### DECT protocol and image reconstruction

An advanced DECT scanner was used for routine clinical practice (SOMATOM Force, Siemens Healthineers, Forchheim, Germany; Revolution HD GSI; GE Healthcare, Waukesha, WI, USA). The detailed CT protocol is presented in Table 1. To display IC on DECT images, commercially available software (syngo.via, version VB40A; Siemens Healthineers, and AW VolumeShare 7, GE Healthcare) was used along with an iodine subtraction algorithm (Liver VNC; Siemens Healthineers, and GSI Viewer, GE Healthcare). Two radiologists (with 5 and 10 years of experience in abdominal radiology, respectively, and 3 years of experience in DECT studies) independently measured the ICs in 2 contrast-enhanced phases of abdominal radiography. They were blinded to histologic and postoperative results and followed a consensus. The region of interest (ROI) was drawn along the exact tumour margin. To avoid blood vessels and bile ducts at the same level in the tumour-free liver parenchyma and calculate the average IC in the liver parenchyma, 3 additional ROIs measuring approximately 1 cm in diameter were constructed. At the level of tumour ROI measurement, IC was measured within the abdominal aorta to calculate normalized IC (NIC), as NIC = IC_tumour_/IC_aorta_. All statistical data were presented as mean values.

**Table 1.** CT protocols.

Scan parameters	CT scanner							
	Centre 1: SOMATOM Force, Siemens Healthineers				Centre 2: Revolution HD GSI, GE Healthcare			
	No contrast	Arterial phase	Portal phase	Delayed phase	No contrast	Arterial phase	Portal phase	Delayed phase
Tube voltage (kV)	120	100/Sn150	100/Sn150	120	120	80/140	80/140	120
Tube current (mAs)	Quality reference 147	Quality reference 210/105	Quality reference 210/105	Quality reference 147	SmartmA 200-300	GSI Assist 200	GSI Assist 200	SmartmA 200-300
Collimation (mm)	57.6	57.6	57.6	57.6	80	80	80	80
Pitch factor	0.6	0.6	0.6	0.6	0.992	0.992	0.992	0.992
Reconstructed section thickness (mm)	5 mm/1 mm	5 mm/1 mm	5 mm/1 mm	5 mm/1 mm	5 mm/1 mm	5 mm/1 mm	5 mm/1 mm	5 mm/1 mm
Reconstructed section spacing (mm)	5 mm/1 mm	5 mm/1 mm	5 mm/1 mm	5 mm/1 mm	5 mm/1 mm	5 mm/1 mm	5 mm/1 mm	5 mm/1 mm
Rotation time (s)	0.5	0.5	0.5	0.5	0.5	0.5	0.5	0.5
Reconstruction algorithms	ADMIRE strength 3	ADMIRE strength 3	ADMIRE strength 3	ADMIRE strength 3	ASIR-V 40%	ASIR-V 40%	ASIR-V 40%	ASIR-V 40%

Injection protocol: intravenous injection of isotonic nonionic iodine contrast agent (iodixanol 320 mg iodine/mL) 1.5 mL/kg. The contrast medium was injected at the rate of 3.0 mL/s, then followed by 40 mL saline. After no contrast scan, there is an arterial DECT phase (using bolus tracking technique), a portal DECT phase (20 s), and a delayed phase CT (2 min) following the attenuation threshold for 120 HU in the abdominal aorta.

Abbreviations: ADMIRE = advanced modelled iterative reconstruction; ASIR = adaptive statistical iterative reconstruction; GSI = gemstone spectral imaging.

### Histopathology

To identify the presence of MVI, pathologists from the same university pathology centre examined all surgical specimens at 2 centres and reached a consensus. MVI is defined as a nest of >50 cancer cells observed microscopically in the lumen of a vessel lined with endothelial cells.^19^

### Statistical analysis

Based on the primary data, 2-sample *t*-test are used for normally distributed continuous variables, and Mann-Whitney *U* test are used for non-normally distributed variables, and Pearson’s χ^2^ test is for categorical variables. To determine whether PCA is necessary, we analysed the information redundancy status among DECT parameters and laboratory results of the primary dataset using Kaiser-Meyer-Olkin factor adequacy and Bartlett’s spherical test. Then, PCA of all continuous variables (quantitative DECT parameters and laboratory results) was performed. An oblimin rotation was used to facilitate the interpretation, and a scree plot was constructed to filter the fundamental components. The selected principal components (PCs) should cumulatively account for at least 60% of the overall variance in the data. Then, the loading matrix obtained from PCA analysis was also applied to the validation dataset for component extraction. To evaluate whether the obtained PCs showed significant differences across different MVI groups, Mann-Whitney *U* test was conducted for the PC scores of the primary and validation datasets. For example, if *X*_1_, *X*_2_, *X*_3_, …, *X_n_* are the original variables, and *V*_1_, *V*_2_, *V*_3_, …, *V_n_* are the loadings of the first principal component, then the PC score for the first component (PC1) for an observation with standardized values *Z*_1_, *Z*_2_, *Z*_3_, …, *Z_n_*, would be calculated as (*n* is the total number of features):

$$\text{PC}1=Z_{1}\;\times \;V_{1}\;+\;Z_{2}\;\times \;V_{2}\;+\;Z_{3}\;\times \;V_{3}\;+\;\cdots \;+\;Z_{n}\;\times \;V_{n}.$$

Similarly, PC scores for other components can be calculated using their corresponding loadings and standardized values of the original variables. The factor loading weighting for the PC1-4 is located in the columns of Table 4. To quantify the predictive ability of the obtained PCs for MVI, a general linear classifier was applied to each PC in the primary and validation datasets. The receiver operating characteristic curve (ROC) value was calculated for each component. For each PCA component, we sorted the PC scores of all subjects from small to large and used them as classification thresholds. To draw the ROC curve, we divide the samples into positive and negative classes according to the PC score and classification threshold and calculate the true-positive rate and false-positive rate under this classification threshold. Performance was evaluated using the area under the ROC curve (AUC).

**Table 2.** Patient characteristics and laboratory results.

Variable	Primary data (*n* = 82)		*P*-value	Validation data (*n* = 37)		*P*-value
	Negative	Positive		Negative	Positive	
**Age**	57.63 ± 10.08	58.30 ± 10.23	.775	55.00 ± 2.61	56.13 ± 1.76	.712
**Sex**			.490			.265
Female	12/52	5/30		3/14	9/23	
Male	40/52	25/30		11/14	14/23	
**Hepatitis**			.921			.225
None	7/52	5/30		4/14	2/23	
Hepatitis B	43/52	24/30		10/14	20/23	
Others	2/52	1/30		0/14	1/23	
**Cirrhotic**			.018^a^			.042^a^
Yes	28/52	24/30		9/14	21/23	
None	24/52	6/30		5/14	2/23	
**AFP (ng/mL)**	13.35 [3.40-346.20]	345.90 [9.88-610.72]	.016^a^	4.15 [2.02-18.03]	55.10 [3.75-625.30]	.009^a^
**γ-GGT (U/L)**	44.50 [20.75-72.25]	61.50 [40.00-84.50]	.055	42.50 [21.25-52.50]	58.00 [36.00-79.50]	.216
**TBIL (µmol/L)**	16.15 [11.45-20.30]	15.55 [12.08-17.65]	.386	14.90 [10.25-22.95]	11.70 [10.30-16.30]	.355
**DBIL (µmol/L)**	4.75 [2.68-7.93]	3.90 [2.70-6.10]	.435	6.05 [1.90-9.07]	3.60 [2.35-4.75]	.287
**IBIL (µmol/L)**	11.45 [7.97-14.05]	10.50 [9.12-13.23]	.661	11.55 [8.60-14.97]	10.10 [7.50-13.85]	.661
**ALT (U/L)**	32.00 [22.75-53.75]	35.50 [24.00-81.75]	.238	30.50 [25.25-65.25]	39.00 [22.50-59.00]	.988
**AST (U/L)**	33.00 [25.75-54.50]	38.00 [28.00-51.00]	.381	32.00 [25.75-35.50]	33.00 [23.50-46.50]	.695
**LDH (U/L)**	197.00 [168.75-218.25]	186.50 [145.25-211.15]	.301	183.50 [161.50-243.00]	179.00 [142.50-207.50]	.287

Data that fit the normal distribution are described using the mean ± SD, and those that do not fit the normal distribution are described as median and interquartile range [IQR 25%-75%].

a Statistical significant difference was considered at *P *<* *.05.

Abbreviations: γ-GGT = gamma-glutamyltransferase; AFP = alpha-fetoprotein; ALT = alanine aminotransferase; AST = aspartate aminotransferase; DBIL = direct bilirubin; IBIL = indirect bilirubin; LDH = lactate dehydrogenase; TBIL = total bilirubin.

**Table 3.** DECT quantitative parameters.

DECT quantitative parameters	Primary data (*n* = 82)		*P*-value	Validation data (*n* = 37)		*P*-value
	Negative	Positive		Negative	Positive	
Tumour diameter (mm)	40.61 [29.96-57.16]	45.82 [32.54-61.18]	.381	39.30 [29.45-47.48]	44.70 [31.93-58.30]	.222
ICa (mg/mL)	1.40 ± 0.64	1.48 ± 0.41	.485	1.88 ± 0.22	1.87 ± 0.39	.989
NICa (×100%)	14.21 ± 5.47	11.25 ± 3.37	.003^a^	16.25 ± 1.27	10.90 ± 0.74	.000^a^
ICa/ICaliver	3.11 [2.16-6.97]	3.49 [2.65-7.63]	.317	4.07 [2.92-4.89]	5.50 [2.70-10.50]	.661
ΔICa (mg/mL)	0.98 [0.54-1.17]	1.00 [0.87-1.38]	.220	1.16 [0.90-1.47]	1.01 [0.97-1.54]	.925
ICp (mg/mL)	2.08 [1.65-2.30]	1.85 [1.60-2.03]	.143	2.20 [1.91-2.48]	1.86 [1.75-2.45]	.309
NICp (×100%)	41.9 [31.6-54.6]	32.2 [28.7-42.8]	.021^a^	46.7 [39.6-55.8]	33.1 [27.2-48.5]	.028^a^
ICp/ICpliver	1.09 [0.84-1.25]	1.00 [0.82-1.25]	.799	1.17 [1.01-1.27]	1.26 [0.84-1.42]	.876
ΔICp (mg/mL)	0.07 ± 0.58	0.04 ± 0.61	.839	0.32 ± 0.13	0.08 ± 0.18	.348

Data that fit the normal distribution are described using the mean ± standard deviation, and those that do not fit the normal distribution are described as median and interquartile range [IQR 25%-75%].

a Statistical significant difference was considered at *P *<* *.05.

Abbreviations: ΔICa = ICa-ICaliver; ΔICp = ICp-ICpliver; ICa = arterial phase iodine concentration of the tumour; ICaliver = arterial phase iodine concentration of the liver parenchyma; ICp = portal phase iodine concentration of the tumour; ICpliver = portal phase iodine concentration of the liver parenchyma; NICa = normalized arterial phase iodine concentration of the tumour; NICp = normalized portal phase iodine concentration of the tumour.

**Table 4.** Factor loading weighting of the first 4 principal components (PC1-PC4), with oblimin rotation (primary data).

Eigenvalues	PC1	PC2	PC3	PC4
Age	0.2885	−0.0799	−0.2768	−0.2714
AFP	0.3853	0.3907	−0.2733	−0.2641
γ-GGT	−0.1284	0.6907^a^	0.2084	0.0731
TBIL	0.0474	0.0422	0.9479^a^	0.019
DBIL	0.0195	0.1382	0.8919^a^	−0.148
IBIL	0.0575	−0.0143	0.8849^a^	0.1079
ALT	0.0215	0.8676^a^	0.0793	−0.0379
AST	0.0566	0.8715^a^	0.0405	−0.0101
LDH	−0.1043	0.4694	0.1737	0.3566
Tumour diameter	0.0928	0.549^a^	−0.3294	0.0976
ICa	0.1157	−0.0312	0.1556	0.8696^a^
NICa	0.3516	−0.3086	0.4452	0.4029
ICa/ICaliver	0.4135	0.0023	−0.3563	0.5607^a^
ΔICa	−0.2097	0.0491	−0.1106	0.8853^a^
ICp	0.9002^a^	0.1643	−0.1743	0.1312
NICp	0.7427^a^	−0.0389	0.1586	0.1684
ICp/ICpliver	0.9155^a^	−0.0502	0.1216	−0.1223
ΔICp	0.9124^a^	−0.0543	0.1146	−0.1151

a An influential factor can be considered highly correlated with a PC when its correlation modulus exceeds 0.5. Correlations among the remaining PCs (5-18) were not examined, as they were excluded from the analysis.

Abbreviations: γ-GGT = gamma-glutamyltransferase; ΔICa = ICa-ICaliver; ΔICp = ICp-ICpliver; AFP = alpha-fetoprotein; ALT = alanine aminotransferase; AST = aspartate aminotransferase; DBIL = direct bilirubin; IBIL = indirect bilirubin; ICa = arterial phase iodine concentration of the tumour; ICaliver = arterial phase iodine concentration of the liver parenchyma; ICp = portal phase iodine concentration of the tumour; ICpliver = portal phase iodine concentration of the liver parenchyma; LDH = lactate dehydrogenase; NICa = normalized arterial phase iodine concentration of the tumour; NICp = normalized portal phase iodine concentration of the tumour; TBIL = total bilirubin.

Statistical analysis was performed and figures were constructed using RStudio for Windows (free version 1.4.1103, https://www.rstudio.com) with the R software packages “psych,” “GPArotation,” “survival,” “PredictABEL,” “pROC,” and “ggplot2” as well as Matlab homemade codes. A *P*-value of <.05 was considered to indicate statistical significance.

## Results

### Patient characteristics and laboratory results

**Figure 1.**
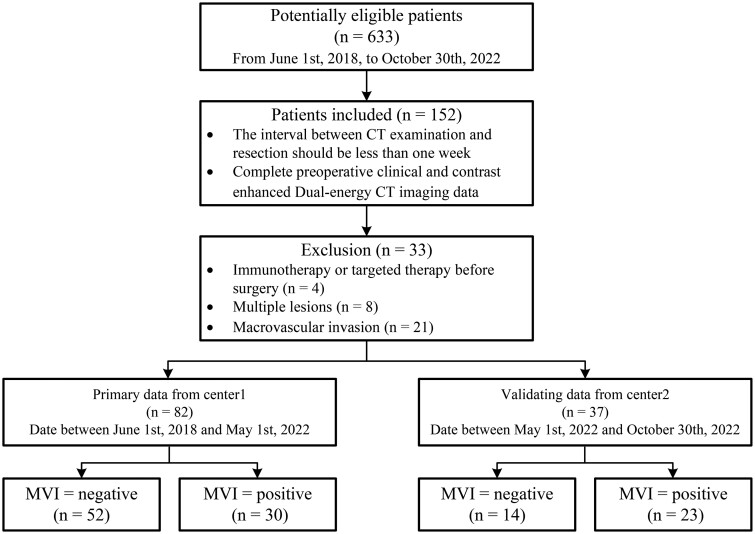
Flowchart shows the study inclusion and exclusion criteria. Abbreviations: DECT = dual-energy CT; MVI = microvascular invasion.

Among the 633 eligible patients, 448 patients had no available DECT data, 152 satisfied the inclusion criteria, and 33 were excluded from this study due to various reasons, such as immunotherapy or targeted therapy before resection (*n* = 4), the presence of multiple lesions (*n* = 8), and the presence of macrovascular invasion (*n* = 21) (Figure 1). A total of 119 patients (median age, 58 years; interquartile range, 51-64 years; 90 men) were included in this study (Table 2). Among them, 53 patients (44.5%) had positive postoperative MVI. The primary and validation datasets included 30 (37%) and 23 (62%) MVIs, respectively. The primary dataset revealed that patients with a history of cirrhosis had a higher probability of occurrence of MVI when HCC occurred (odds ratio 4.27; 95% CI, 1.20-9.78; *P *<* *.05). A significant difference was noted (*P *<* *.05) in the alpha-fetoprotein (AFP) levels between the MVI-negative and MVI-positive groups in both primary (median, 13.35 ng/mL; interquartile range, 3.40-346.20 ng/mL vs. median, 345.90 ng/mL; interquartile range, 9.88-610.72 ng/mL) and validation (median, 4.15 ng/mL; interquartile range, 2.02-18.03 ng/mL vs. median, 55.10 ng/mL; interquartile range, 3.75-625.30 ng/mL) datasets.

### DECT quantitative analysis

**Figure 2.**
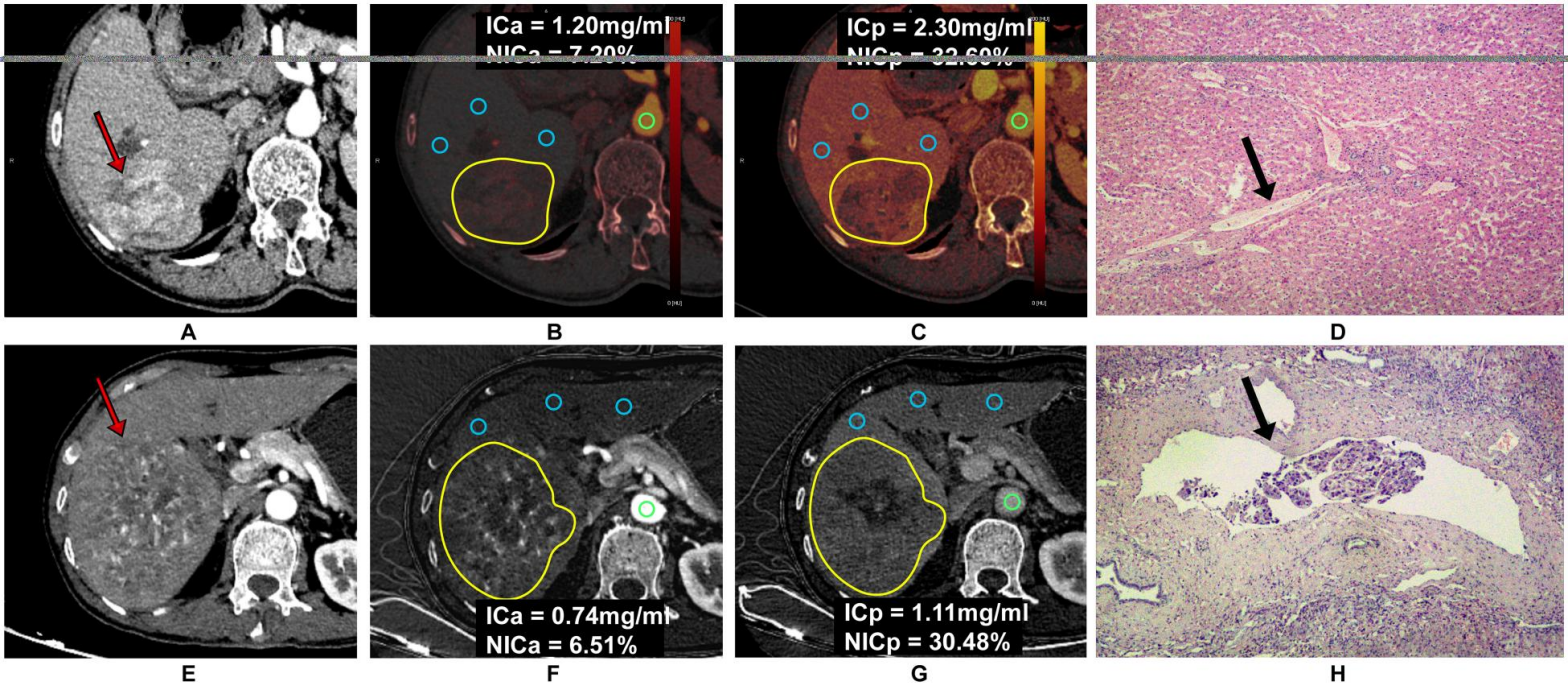
Typical contrast-enhanced dual-energy CT (DECT) images in patients with and without microvascular invasion (MVI). (A) A 69-year-old man with hepatocellular carcinoma (HCC) without MVI; preoperative DECT image of the axial arterial phase (red arrow). (B) Arterial phase iodine overlay image shows that the mean iodine concentration (IC) (yellow outline) in the HCC lesion was 1.20 mg/mL, and the normalized IC was 7.2%. (C) Portal venous phase iodine overlay image shows that the mean IC (yellow outline) was 2.30 mg/mL, and the normalized iodine concentration (NIC) was 32.6%. (D) A postoperative photomicrograph shows no cancer thrombus in the hepatic interlobular vein, indicating MVI negativity (black arrow) (haematoxylin-eosin stain; primary magnification, ×40). (E) A 69-year-old man with HCC and MVI; preoperative DECT image of the axial arterial phase (red arrow). (F) Arterial phase iodine overlay image shows that the mean IC (yellow outline) in the HCC lesion was 0.74 mg/mL, and the normalized IC was 6.5%. (G) Portal venous phase iodine overlay image shows that the mean IC (yellow outline) was 1.11 mg/mL, and the NIC was 30.5%. (H) A postoperative photomicrograph shows a cancer thrombus in the hepatic interlobular vein within a distance of <1 cm adjacent to the cancer (black arrow) (haematoxylin-eosin stain; primary magnification, ×40). The ROIs with blue colour were placed in the tumour-free liver parenchyma to avoid blood vessels and bile ducts at the same level and determine the average IC of the liver parenchyma. To calculate the NIC, the ROI with green colour was placed on the same plane as the abdominal aorta. Arterial phase IC (ICa), portal phase IC (ICp), normalized arterial phase IC (NICa), normalized portal phase IC (NICp).

For both tumour and tumour-free liver parenchyma, 9 quantitative DECT parameters were analysed, as shown in Table 3. The MVI-free and MVI-positive DECT images of 2 patients are presented in Figure 2. The results indicated that compared with the MVI-negative group, the normalized arterial phase IC (NICa) and normalized portal phase IC (NICp) in the tumour were significantly lower in the MVI-positive group in both the primary (median, 10.5%; interquartile range, 9.6%-13.5% vs median, 13.2%; interquartile range, 10.7%-18.5%, and median, 32.2%; interquartile range, 28.7%-42.8% vs median, 41.9%; interquartile range, 31.6%-54.6%, respectively, *P *<* *.05) and validation (median, 10.5%; interquartile range, 8.2%-12.0% vs median, 16.1%; interquartile range, 13.5%-17.5%, and median, 33.1%; interquartile range, 27.2%-48.5% vs median, 46.7%; interquartile range, 39.6%-55.8%, respectively, *P *<* *.05) datasets.

### Principal component analysis

The primary dataset underwent KMO test and Bartlett’s spherical test, yielding a coefficient of 0.56 and a *P*-value of <2.2e−16, respectively. These results demonstrated the existence of significant information redundancy among the features and supported the use of PCA. Using all 18 consecutive data points, the eigenvalues and eigenvectors were calculated to determine the first 4 PCs, which accounted for 67.9% of the variance in the data. In particular, the loadings of 24.1, 16, 15.4, and 12.4 were recorded for PC1, PC2, PC3, and PC4, respectively (Figure S1). The factor loading weights for oblimin rotations of these components are shown in Table 4.

PC1 contains information regarding the preoperative portal phase IC of the tumour (ICp) associated with MVI (Table 3), as indicated by the high loadings for ICp/ICpliver (0.9155), ΔICp (0.9124), ICp (0.9002), and NICp (0.7427). Similarly, PC2 correlates with tumour diameter (0.549) and hepatocyte damage indicators such as aspartate aminotransferase (0.8715), alanine aminotransferase (0.8676), and gamma-glutamyltransferase (0.6907). PC3 primarily comprises liver function markers, with high loadings for total bilirubin (TBIL; 0.9479), direct bilirubin (DBIL; 0.8919), and indirect bilirubin (IBIL; 0.8849). PC4 reflects preoperative ICa associated with MVI in HCC, as indicated by loadings for ΔICa (0.8853), ICa (0.8696), and ICa/ICaliver (0.5607).

### Mann-Whitney *U* test and ROC curve

**Figure 3.**
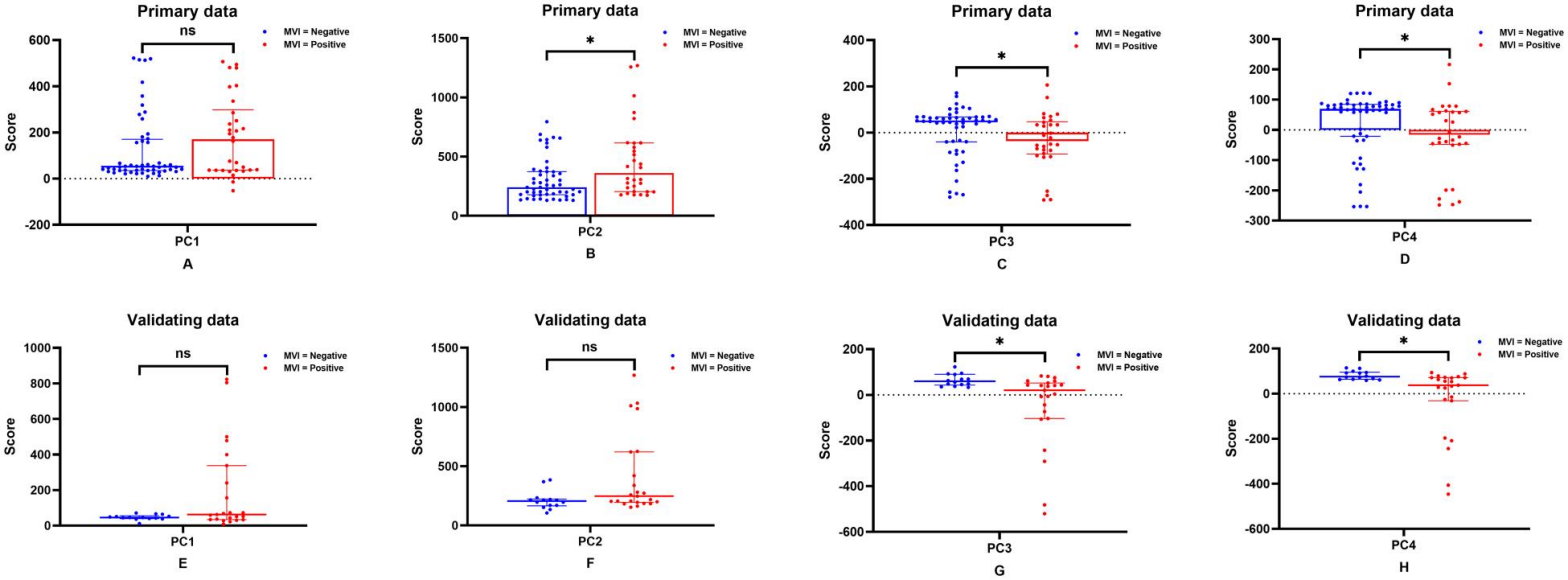
The scatter dot plot shows the representation of individual PCs and MVI groups in both the primary (A-D) and validation (E-H) datasets. The blue plotted points represent the mapping results for each individual on PCs belonging to the MVI-negative group. Conversely, the red points represent the mapping results for each individual on PCs from the MVI-positive group. Additionally, the median is indicated by a line that spans across the graph, indicating the interquartile range. *Significant difference using Mann-Whitney *U* test was considered at *P* < .05. Abbreviation: ns = no significant difference.

**Figure 4.**
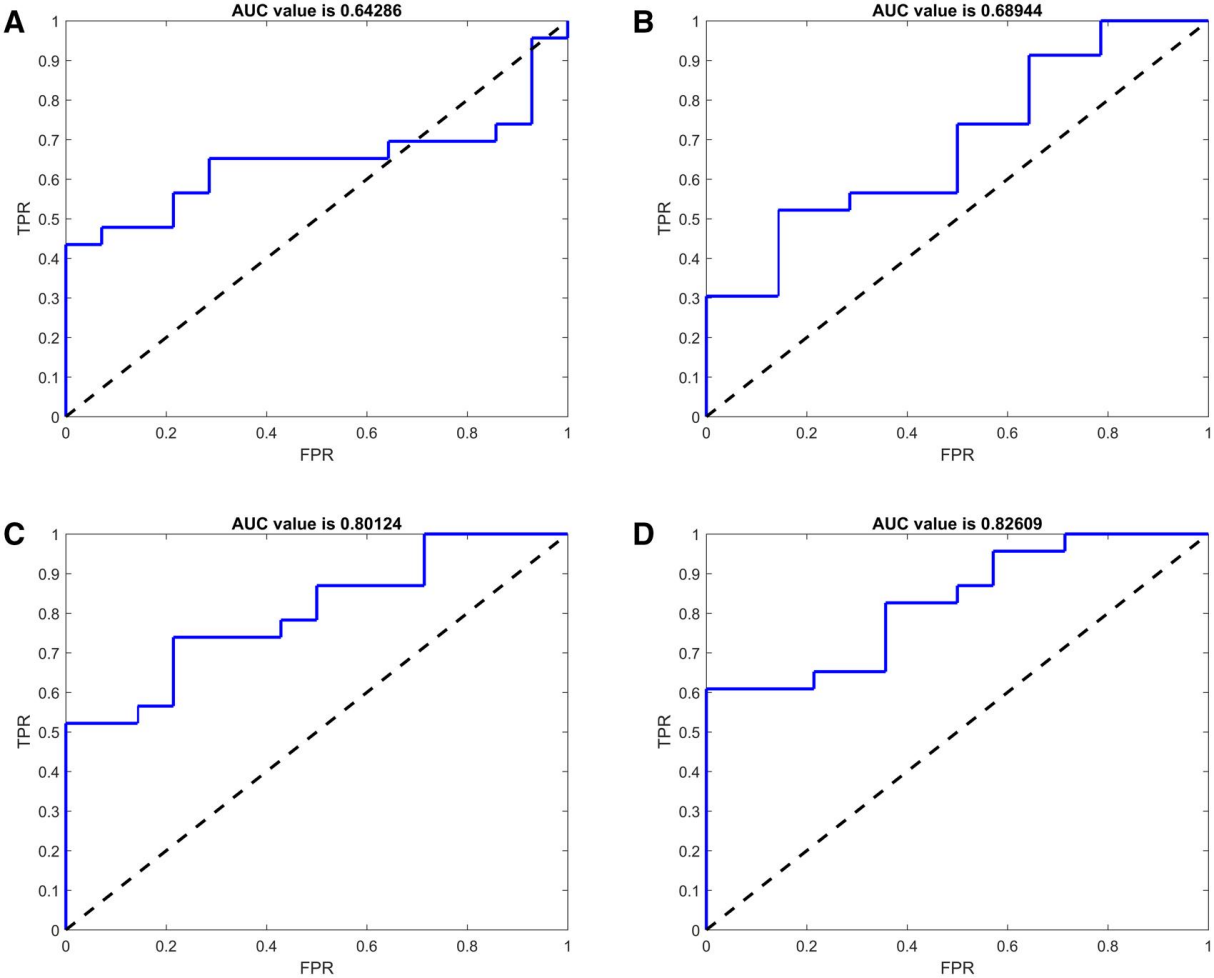
The receiver operating characteristic (ROC) curves of principal component (PC)1-PC4 (A-D) in the validation dataset yielded area under the ROC curve values of 0.643, 0.689, 0.801, and 0.826, respectively. Abbreviations: AUC = area under the curve; TPR = true-positive rate; FRP = false-positive rate.

To evaluate the generalizability of PCA results, Mann-Whitney *U* test was performed in the primary and validation datasets to compare individual PCs between MVI-positive and MVI-negative groups (Figure 3). The results showed significant differences (*P *<* *.05) in PC3 and PC4 between MVI-negative and MVI-positive groups in both datasets. To further assess MVI classification performance in the validation dataset, ROC curves were constructed for all 4 PCs (Figure 4), and their AUC values were calculated as 0.643 (95% CI, 0.463-0.823), 0.689 (95% CI, 0.513-0.862), 0.801 (95% CI, 0.662-0.941), and 0.826 (95% CI, 0.696-0.957), respectively. Based on these AUC values, PC3 and PC4 exhibited a strong potential for accurately classifying MVI, reflecting their ability to provide information regarding MVI status.

## Discussion

Overall survival in patients with HCC has remarkably improved owing to the recent advances in imaging diagnosis, surgical techniques, targeted therapies, and immunotherapy.^20–24^ Despite these advancements, newly diagnosed patients with HCC and MVI present low 5-year survival rates. MVI is only found in malignant tumours, as confirmed via postoperative pathology, with incidence ranging from 15.0% to 57.1%.^3^ In this study, patient characteristics indicated that individuals with a history of cirrhosis and higher levels of AFP had a greater risk of MVI (odds ratio 4.27; 95% CI, 1.20-9.78; *P *<* *.05), which is consistent with previous studies.^25–27^

Although conventional CT signs such as nonsmooth boundaries, hypodense halos, and visible intratumoural arteries may suggest the presence of MVI, the diagnostic accuracy relies on image quality and radiologist expertise. MVI prediction based on deep learning methods is considered a reliable method,^10^^,^^28^^–^^30^ and such methods are becoming increasingly well-established, however, clear interpretable quantitative metrics are still required. Recent studies have shown that quantitative IC plays an important role in tumour diagnosis. Fan et al closely reviewed relevant literature published in the past decade to explore the clinical value of DECT in the context of HCCs and concluded that standardized quantitative parameters derived from DECT can be a useful tool for HCC surveillance.^31^ Our DECT IC result revealed that the MVI-positive group had lower NICa and NICp values than the MVI-negative group (*P *<* *.05). These findings provide important insights into preoperative MVI risk assessment. However, the present study results contradict those reported by Kim et al,^32^ which revealed that HCCs with MVI had significantly higher NICs than those without MVI (*P *<* *.05). Still, Yang et al suggested that the MVI-positive group had a higher incidence of necrosis,^33^ potentially resulting in lower NICa and NICp values. These contradictory findings may be attributed to the differences in the sampling design, which require further investigation.

Most previous studies have chosen to use supervised learning for modelling, which may lead to risks of data dimensionality disaster, low model calculation efficiency and overfitting. To solve this problem and help understand the data through discovering hidden structures and patterns in the data, we tried to use PCA in unsupervised learning to solve these problems. PCA can retain the original data information to the greatest extent to achieve dimensionality reduction, while outputting a linear combination of the original features. This combination ensures that the selected PCs can effectively represent the original data, which is helpful for improving the generalization ability and robustness of the model of the model.

Utilizing PCA, we effectively reduced data dimensionality, discerning 4 PCs comprising differently weighted feature combinations. Collectively, these components explained 67.9% of the variance in the original dataset. Various features in the original data undergo reweighting based on the distinct weights assigned by these 4 PCs, resulting in the derivation of the corresponding principal component scores. The results revealed significant differences (*P* < .05) in PC3 and PC4 across MVI groups in both the primary and validation datasets. Furthermore, PC3 and PC4 exhibited superior performance in MVI classification, with AUC values of 0.8410 and 0.8373, respectively. Moreover, bilirubin (TBIL, DBIL, and IBIL) was significantly correlated with PC3 (with factor loading weights of 0.9479, 0.8919, and 0.8849, respectively). Elevated bilirubin levels are usually indicative of abnormal liver function, and as liver cancer progresses, bilirubin dysregulation occurs. Carr et al analysed a cohort of 2416 patients with HCC and found that patients with higher bilirubin levels had poorer prognosis and indices of aggressiveness, confirming the significance of bilirubin in HCC.^34^ Chan et al identified high albumin bilirubin grading as a key parameter associated with early recurrence and developed a relevant preoperative model to predict the risk of early recurrence after HCC surgical resection.^35^ Several recent studies have shown that nomogram models combined with TBIL provide better predictive performance in predicting MVI,^26^^,^^36^^,^^38^ but the mechanism of interaction between bilirubin levels and MVI remains unclear. We hypothesized that MVI leads to local tissue oedema and limits the local function of the small bile ducts. Also, ICa, ICa/ICaliver, and ΔICa were significantly correlated with PC4 (with factor loading weights of 0.8853, 0.8696, and 0.5607, respectively). The primary characteristic of typical HCC, as defined by the Liver Imaging Reporting and Data System criteria, is nonrim hyperenhancement in the arterial phase compared to normal liver tissue. This indicates that HCC is a type of arterial phase hyperintense blood-rich tumour. DECT parameters such as ICa, ICa/ICaliver, and ΔICa reflect the iodine content in arterial phase lesions from different perspectives and are positively correlated with the degree of enhancement of arterial phase lesions. Zhang et al concluded that arterial enhancement fraction (AEF) and hepatic artery perfusion index (HPI) were independent risk factors for predicting MVI by analysing perfusion and related parameters in 3-phase enhancement scans. They found that the 2 parameters related to HPI (ΔHPI(Max) and relative-HPI(Max)) had the highest sensitivity for predicting MVI at 0.854.^38^ These findings are similar to ours, as ICa, ICa/ICaliver, and ΔICa respond to arterial phase blood supply similarly to AEF and HPI. Studies have shown that HCC with cirrhosis leads to a decrease in portal venous pressure (PVP). To compensate for this decrease, arterial liver perfusion increases,^39^ a mechanism known as the hepatic arterial buffer response.^40^ This eventually leads to an increase in ICa, ICa/ICaliver, and ΔICa. Similarly, MVI involves small portal vessels causing a decrease in PVP, resulting in increased ICa/ICaliver and ΔICa.

This study has several limitations. First, this retrospective study involved 2 centres and lacked random data splitting for training and validation owing to the small sample size. Moreover, the 2 centres used different DECT imaging systems, which may have affected the results (centre 1: dual tubes with beam filtration, centre 2: rapid voltage switching with a single tube). However, regardless of the technology used for data acquisition, all commercial multienergy CT systems provide DECT data.^41^^,^^42^ Based on the recent study comparing the quantitative accuracy of different DECT scanners, firstly, the third-generation DECT scanners analysed in this study are statistically ranked in the first tier for accuracy of IC. Secondly, the protocols used in centre 1 and centre 2 demonstrate similar mean absolute percentage errors, approximately 4.72% ± 3.31% and 4.68% ± 3.43%, respectively.^43^ However, due to the potential differences in measurements, it is suggested that future prospective studies with a larger sample size be conducted. These studies may also consider incorporating additional types of DECT devices to further investigate and validate these findings. Maybe Dual source concepts with photon counting detectors could potentially serve as a solution to address this issue. Second, instead of volumetric iodine quantification, 2-dimensional ROIs were used in this study; therefore, it is necessary to confirm the difference between volumetric and 2-dimensional measurements. Finally, morphological assessments other than tumour size were not included in our study. Further investigation is warranted to determine the potential benefits of combining quantitative data with morphological parameters for the preoperative prediction of MVI.

## Conclusion

Our study demonstrates that 2 PCs with oblimin rotations can provide valid information for the preoperative classification of MVI. The bilirubin parameters (TBIL, DBIL, and IBIL) and ICa in the tumour were considered important for MVI analysis. The combination of DECT IC and laboratory features based on varying factor loadings can potentially assist surgeons in discerning high-risk patients for MVI.

## Supplementary Material

tqae116_Supplementary_Data
