# Supplementary material for: Dual-energy computed tomography iodine quantification combined with laboratory data for predicting microvascular invasion in hepatocellular carcinoma: a two-centre study
Source: Br J Radiol. 2024 Jun 13;97(1160):1467–75. doi: 10.1093/bjr/tqae116 (PMC11256957; doi:10.1093/bjr/tqae116)
Supplement: tqae116_Supplementary_Data [file tqae116_supplementary_data.docx]

**SUPPLEMENTARY**

**
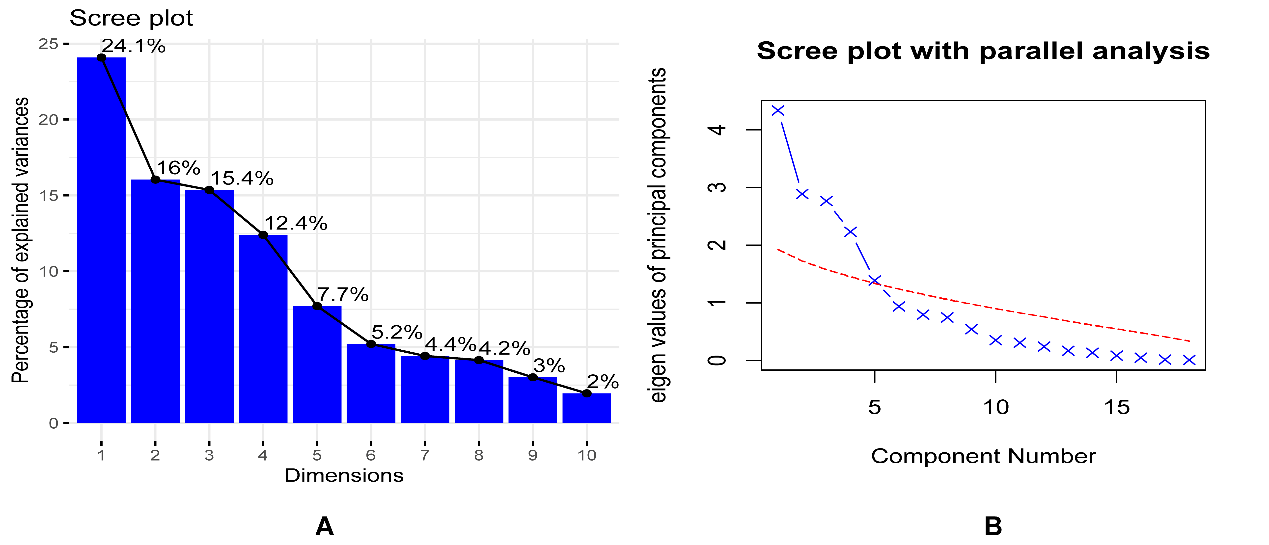
**

**Supplementary Figure 1.** The scree plot in Figure A shows 10 principal components (PC1 to PC10) along with the corresponding percentage of explained variance for each component. Specifically, PC1 to PC4 had loadings of 24.1%, 16%, 15.4%, and 12.4%, respectively. Figure B presents the scree plot with parallel analysis of PCs, displaying the straight line with x symbols for the screen plot and the dotted line for the parallel analysis of 100 simulations. The results indicate that retaining the first four principal components is sufficient to preserve most of the information present in the dataset.
